# Presenting Phenotypes and Early Diagnostic Approach in Immune Thrombotic Thrombocytopenic Purpura: A Narrative Review for Emergency and Acute Care Practice

**DOI:** 10.3390/life16101591

**Published:** 2026-09-23

**Authors:** Cătălin-Alexandru Anghel, Cristina Mambet, Alina Mititelu, Silvia Nica, Ana-Maria Vlădăreanu

**Affiliations:** 1 Doctoral School, Carol Davila University of Medicine and Pharmacy, 050474 Bucharest, Romania; anamaria.vladareanu@umfcd.ro; 2 Department of Hematology, Carol Davila University of Medicine and Pharmacy, Emergency University Hospital of Bucharest, 050098 Bucharest, Romania; cristina.mambet@umfcd.ro (C.M.); alina.mititelu@umfcd.ro (A.M.); 3 Department of Emergency and First Aid, Carol Davila University of Medicine and Pharmacy, Emergency University Hospital Bucharest, 050474 Bucharest, Romania; silvia.nica@umfcd.ro

**Keywords:** immune thrombotic thrombocytopenic purpura, thrombotic microangiopathy, ADAMTS13 deficiency, microangiopathic hemolytic anemia, PLASMIC score, French score, emergency medicine, diagnostic delay, early recognition

## Abstract

Immune thrombotic thrombocytopenic purpura (iTTP) is a rare condition with multisystem involvement that, in the absence of early recognition and prompt initiation of specific treatment, can be life-threatening. Nowadays, the diagnosis of this pathology represents a challenge in itself, determined by the association of several factors: the heterogeneity of the clinical picture, the frequent absence of all components of the classic pentad, as well as the overlap of neurological, cardiac, abdominal, or renal manifestations with those of much more common medical emergencies. This narrative review synthesizes the current data from the literature on the presentation of iTTP in emergency departments, the clinical and paraclinical elements that outline the clinical picture and support the clinical suspicion, the role of ADAMTS13 activity, and the usefulness of integrating the PLASMIC and French scores in current practice and in therapeutic decision making. The available data highlight the association between thrombocytopenia and microangiopathic hemolytic anemia as a central element of suspicion and support integrating organ involvement assessment, differential diagnosis, and early sampling for ADAMTS13 testing into the evaluation of patients with suspected iTTP. It should also be noted that in patients with high clinical probability of iTTP, the decision to initiate specialized treatment should not be postponed until the ADAMTS13 result is obtained. In this way, a dynamic approach, based on the corroboration of data obtained from the medical history, clinical examination, laboratory and diagnostic investigations, and probability assessment tools, facilitates early diagnosis and rapid access to specific therapy with a favorable impact on the prognosis.

## 1. Thrombotic Thrombocytopenic Purpura as a Diagnostic Emergency in Modern Medicine

Immune thrombotic thrombocytopenic purpura (iTTP) is a systemic disease with multiorgan involvement, representing a severe thrombotic microangiopathy clinically defined by the association of thrombocytopenia and microangiopathic hemolytic anemia (MAHA). This autoimmune-mediated disease is associated with an acquired deficiency in ADAMTS13 (a disintegrin and metalloproteinase with thrombospondin type 1 motif, member 13) activity. In the setting of this deficiency, ultra-large multimers of von Willebrand factor persist and accumulate in the bloodstream, promoting spontaneous platelet aggregation, which contributes to the formation of disseminated microthrombi that occlude microcirculation [1,2,3]. The immediate clinical consequences are massive platelet consumption and mechanical shearing of red blood cells, culminating in acute organ ischemia [4]. Despite the fact that this enzymatic deficiency is a necessary condition for disease onset, current epidemiological evidence indicates that the condition has a particular profile, with an additional triggering factor often required for the disease to become clinically manifest.

At the population level, iTTP is a rare condition, with an estimated annual incidence of between two and six cases per million inhabitants [1,5]. Analysis of the distribution by age group and sex outlines the profile of the typical patient: the disease predominantly affects adults (with a median age at onset of around 40 years), with a marked predilection for the female sex (women are affected three times more frequently than men and account for up to 77% of the cases included in large registries) [6,7]. In addition, the disease shows a racial predisposition, with a much higher incidence among people of African origin, which may increase exponentially when associated with the female sex [1,3]. Beyond these demographic characteristics, an additional triggering factor is present in most cases and may include: infections (influenza virus [8], COVID-19 [9,10], HIV, etc.), autoimmune diseases (frequently systemic lupus erythematosus and antiphospholipid syndrome), pregnancy, disseminated malignancy, transplantation, or certain medications (e.g., ticlopidine, quinine, gemcitabine, and calcineurin inhibitors) [6].

Considering the accelerated dynamics of the underlying pathophysiological process, multiple systemic consequences develop within a relatively short time. In this context, iTTP represents one of the most dramatic hematological emergencies and, in the absence of treatment, is associated with a mortality rate exceeding 90% [1]. Therapeutic advances over recent decades have transformed this disease from an almost invariably fatal condition into a treatable one. Thus, the introduction of therapeutic plasma exchange represented a defining step in the evolution of treatment, initially reducing mortality to 9–20%. Subsequently, the addition of immunomodulators increased survival to over 90% [1,11,12]. In addition, the HERCULES study, one of the most important modern trials in iTTP, changed the standard of care through the addition of caplacizumab. As a result, the rates of refractory disease and mortality decreased to 2.2% [13,14].

Despite all the significant therapeutic advances, the prognosis of patients with iTTP remains dependent on the timeliness of treatment initiation. Thus, the main determinant of survival remains early diagnosis, followed by admission to the intensive care unit and the urgent initiation of PEX [15]. In current practice, however, the rarity of the disease contributes to diagnostic delays. According to data from the literature, a 24 h delay is frequently associated with a significant increase in the risk of severe neurological events, such as stroke, seizures, or coma (occurring in 67% of patients when therapy is delayed by 24 h, compared with 30% when treatment is initiated within the first 24 h) [16]. Moreover, at the time of presentation, the severity of organ damage, objectively assessed by elevated markers such as lactate dehydrogenase (LDH) above 1500 U/L or elevated troponin levels, is directly correlated with an increased risk of death [17].

The rapid evolution of the clinical picture turns iTTP into a race against time, in which any delay can have major consequences, and treatment initiation cannot wait for diagnostic confirmation through the measurement of ADAMTS13 activity. For this reason, emergency medicine must adopt a modern, proactive, and empirical strategy in which treatment is instituted based on clinical suspicion and clinical probability scores (e.g., the PLASMIC score and the French score). In this way, the life-saving therapeutic sequence can be initiated promptly, and the case can be managed by a multidisciplinary team coordinated by the hematology service.

## 2. From the Classical Pentad to Modern Early Recognition Paradigms

Traditionally, before effective treatment became available, the diagnosis of TTP was based on the clinical recognition of a classic pentad: microangiopathic hemolytic anemia, thrombocytopenia, fever, neurological involvement, and renal involvement [18,19]. These five signs described the natural course of the disease at a time when no treatment was available, reflecting the terminal stage and its progressive evolution toward death [18]. The clinical reality of recent decades has shown that waiting for the appearance of the clinical picture fully defined by the classic pentad in the emergency setting can be a trap. Today, we know that fewer than 7–10% of patients present to the emergency department (ED) or hematology departments with all five elements of the pentad [19]. In addition to the severe neurological consequences of delayed diagnosis, a single-center retrospective study conducted at a university center in Beijing and published in 2025 showed that the presence of all elements of the classical pentad was an independent predictor of mortality (HR 3.5, 95% CI: 1.5–8.4) [16]. These findings reinforce the importance of time as a critical link in the chain of survival. Therefore, waiting for the complete diagnostic pentad may have severe clinical consequences, supporting the need to guide clinicians towards approaches that allow early diagnosis. Thus, current international guidelines recommend considering iTTP in patients presenting with the dyad of unexplained thrombocytopenia and MAHA, without waiting for the manifestation of other classic elements [19,20]. The recognition of this dyad and the use of clinical probability scores may currently provide sufficient clinical criteria for the emergency physician to request a hematology consultation with the aim of initiating early therapy in accordance with international treatment standards [21,22].

## 3. Heterogeneity of Clinical Presentation and Its Impact on Diagnosis

In current medical practice, iTTP behaves as a true “great imitator,” characterized by an extremely polymorphic clinical presentation. This is due to disseminated microvascular ischemia, which favors the development of acute systemic manifestations, but sometimes it might be isolated to a single organ. Data from the literature show that neurological symptoms dominate the initial clinical presentation (39–80% of cases), followed by abdominal manifestations (35–39% of cases) [11,23,24]. In this way, the clinical presentation may closely mimic several clinical entities that are encountered much more frequently in the emergency department, such as ischemic stroke (even with no abnormalities on imaging investigations at presentation) or acute coronary syndrome [25]. By creating the illusion of a localized disease, iTTP establishes, from the time of presentation, the premises for an erroneous clinical interpretation.

This presentation, apparently suggestive of a common disease, predisposes, in such a situation, to the development of cognitive bias mechanisms, particularly diagnostic anchoring and premature closure of the decision-making process. In the current context of emergency departments, where there is a high volume of patients and an accelerated pace of decision making, time frequently becomes the factor that limits detailed clinical assessment and, implicitly, the complete exploration of the differential diagnosis. Thus, when the clinical picture is highly suggestive of an organ-specific disease, such as ischemic stroke, for which time is also a major determinant of therapeutic success, standardized “fast-track” protocols are inevitably set in motion, generating a genuine “diagnostic tunnel” effect that delays the recognition of iTTP [19]. In this context, the clinician’s attention in the emergency department is often focused on the therapeutic window specific to these protocols, which may lead to insufficient consideration of the information provided by basic investigations, such as the complete blood count. Moreover, severe thrombocytopenia may thus be classified merely as a contraindication to revascularization therapy, rather than as a major indicator of an underlying systemic microangiopathy.

The consequences of this chain of cognitive errors in everyday clinical practice are outlined by data from large national registries. A French study that analyzed a cohort of 423 patients diagnosed with iTTP showed that 20% of them were initially misdiagnosed. Subsequently, subgroup analysis showed that the main diagnostic confusions involved immune thrombocytopenia associated with hemolytic anemia (in 51% of cases) or without hemolytic anemia (in 37% of cases). The factors associated with these diagnostic confusions included several atypical laboratory parameters at presentation, such as a low schistocyte count, the presence of a positive Coombs test, or a hemoglobin level suggestive of less severe anemia, particularly among female patients with autoimmune disease or antinuclear antibodies [26]. This uncertainty translates directly into a delay in the initiation of specialized, life-saving treatment. According to the UK HaemSTAR national study, conducted in 148 patients treated in 80 hospitals between 2014 and 2019, only 64.9% of patients received therapeutic plasma exchange within the first 24 h, with diagnostic uncertainty being the main reason for this delay [27]. Moreover, 27.8% of the recorded deaths were directly related to delayed treatment initiation [27], making this “tunnel effect” devastating and clearly demonstrating both the fatal consequences of diagnostic anchoring and the need to recognize iTTP in emergency departments.

## 4. Rationale, Objectives, and Review Methodology

### 4.1. Review Methodology

This paper is designed as a narrative review and aims to bring together current data from the literature on thrombotic thrombocytopenic purpura, with an emphasis on the early recognition of this clinical entity in the ED, the initial diagnostic approach, and the prompt activation of local management algorithms by a multidisciplinary team. The primary database used for the literature search was PubMed/MEDLINE. Subsequently, the literature selection was supplemented by targeted searches of the specialized literature and by reviewing the reference lists of publications considered relevant. In this regard, clinical guidelines and recommendations from professional societies were included, as well as cohort studies, national or multicenter registries, retrospective and prospective studies, clinical trials, meta-analyses, recent reviews, and seminal studies on pathophysiology or diagnosis.

Articles published between January 2010 and June 2026 were included, with priority given to studies published in the last decade. A few publications from before 2010 were also included selectively when they were considered to represent key contributions to the definition of the disease, the understanding of its pathophysiological mechanisms, the formulation of diagnostic criteria, or the historical evolution of treatment. The database search was performed using queries that included combinations of terms such as “thrombotic thrombocytopenic purpura”, “TTP”, “immune thrombotic thrombocytopenic purpura”, “iTTP”, “thrombotic microangiopathy”, “ADAMTS13”, “ADAMTS13 deficiency”, “microangiopathic hemolytic anemia”, “PLASMIC score”, and “French score”. To improve the search results within the scope of the review, the listed terms were combined with related term from clinical presentation and emergency medicine, such as “presenting symptoms”, “clinical presentation”, “neurologic manifestations”, “cardiac involvement”, “renal involvement”, “abdominal pain”, “misdiagnosis”, “emergency department”, “emergency medicine”, “therapeutic plasma exchange”, and “caplacizumab”. Synonymous terms were used as alternatives, and searches were organized using Boolean operators (AND/OR), according to the focus of each section.

Based on the findings from the first literature searches, sources were selected according to the objectives of the review, with emphasis placed on publications focusing on early detection of iTTP manifestations, distinguishing iTTP from other thrombotic microangiopathies, evaluating the probability of significant ADAMTS13 deficiency, and managing patients at the start of their care. Conversely, publications that were not directly related to iTTP, articles that had limited relevance to the clinical presentation or initial diagnosis of the condition, and isolated case reports that did not provide any additional or useful information relevant to the objectives of the review were excluded. Because the review is based on a narrative approach, the included literature was synthesized descriptively, with an emphasis on the practical implications for emergency department clinicians, as they are often the patient’s first point of contact with the healthcare system. Therefore, the methodology of the review did not follow a PRISMA-based systematic review protocol, as the goal was to integrate relevant literature in a narrative manner rather than perform a quantitative analysis of the data.

### 4.2. Rationale and Objectives

The need for this narrative review lies in the gap between the apparent simplicity of the current diagnostic criteria and the everyday reality of emergency departments, where decisions are made under time pressure and in the context of a very high patient volume. Thus, the diagnostic process may be influenced by cognitive errors and diagnostic anchoring, which may lead to delayed treatment initiation. In everyday practice, emergency physicians rarely encounter this disease, making iTTP difficult to diagnose in the absence of a high level of clinical suspicion, which reflects the fundamental principle that a disease that is not considered cannot be identified in a timely manner. Although iTTP is an emblematic example of a disease that is lethal in the absence of intervention, with its severity well documented, the current scientific literature remains fragmented between the specialized hematological perspective and the practical reality of emergency departments, where the initial presentation may mask the disease as common clinical phenotypes.

Our review is primarily addressed to physicians involved in the initial assessment of the patient in the emergency department, where iTTP must be recognized as quickly as possible based on clinical and laboratory data, which can often be incomplete or non-specific. Although the topic is relevant to many medical specialties, including hematology and intensive care, the material is structured and oriented toward emergency medicine clinicians. It serves as a targeted clinical synthesis tool, focused on three main directions: early recognition in order to raise clinical suspicion, initial stabilization, and prompt referral to the specialist team.

The main contribution of this paper to the literature consists of reorganizing the available data on iTTP within a practical framework aimed at facilitating the appropriate management of the condition during the initial assessment. The importance of this perspective is further emphasized by the fact that a patient’s first contact with the healthcare system most often takes place in the ED, where the evaluation is performed by clinicians who must promptly recognize the warning signs of a rare but potentially fatal condition. For this reason, physicians working in these settings must be aware of several important clinical and practical aspects: early recognition of the association between MAHA and thrombocytopenia, identification of clinical phenotypes that can mimic common conditions, correct use of clinical probability scores, and rapid activation of the multidisciplinary diagnostic and therapeutic chain, with early involvement of a hematologist. Therefore, the main purpose of this work is not to provide an exhaustive overview of the hematological management of the condition, but to integrate clinical and diagnostic information from the perspective of the initial presentation, so as to serve as a useful tool for physicians in the emergency system. This approach provides physicians working in the ED with clear guidance for recognizing a clinical and laboratory profile suggestive of iTTP in order to promptly initiate a multidisciplinary approach.

In this context, this narrative review aims to provide a comprehensive analysis of the clinical polymorphism of iTTP, highlighting the main presenting phenotypes that may mimic isolated organ-specific diseases. In addition, the main diagnostic pitfalls encountered in current emergency practice are analyzed, together with the roles of clinical probability scores such as PLASMIC and French in rapid risk stratification and early initiation of therapy. The paper also aims to integrate current recommendations into a practical and coherent framework that supports the everyday activity of the multidisciplinary case-management team, activated by the emergency physician but subsequently coordinated by a hematologist experienced in the early recognition and prompt initiation of specific treatment.

### 4.3. Terminological Clarification

To ensure terminological clarity and scientific rigor, we note that in this manuscript the term thrombotic microangiopathy (TMA) is used as an umbrella term for the group of clinical and laboratory syndromes characterized by thrombocytopenia, microangiopathic hemolytic anemia, and organ damage secondary to microvascular thrombosis, including hemolytic uremic syndromes (HUS) and thrombotic thrombocytopenic purpura (TTP) [20]. The latter is a condition that can be classified into a congenital form and an acquired, immune-mediated form called immune thrombotic thrombocytopenic purpura (iTTP). The term “TTP” is used when discussing general aspects of the disease, such as the initial clinical presentation, the urgency of diagnosis, and the need for early recognition, while “iTTP” is used for aspects specific to the acquired form. In the ED, the diagnosis of TTP/iTTP is presumptive and based on clinical and laboratory findings, while confirmation of iTTP is based on demonstrating ADAMTS13 activity below 10% in an appropriate context [22].

## 5. Pathophysiological Framework Relevant to Clinical Presentation

### 5.1. ADAMTS13 Deficiency and Mechanisms of Thrombotic Microangiopathy

The pathophysiology of iTTP is based on the existence of a critical molecular imbalance between von Willebrand factor (vWF) and its cleaving enzyme, ADAMTS13, a metalloprotease whose only known substrate is this factor [28,29]. Normally, endothelial cells release vWF into the circulation in the form of ultra-large multimers (ULvWF). Once in the bloodstream, these multimers are subjected to shear forces that alter their conformation, exposing both the platelet-binding sites and the cleavage site for the enzyme [28,30]. By cleaving these giant multimers, ADAMTS13 prevents spontaneous platelet adhesion and, consequently, the formation of microthrombi in the circulation.

The disruption of this physiological balance occurs with the development of IgG autoantibodies that directly target the ADAMTS13 metalloprotease. These autoantibodies cause a severe deficiency of this enzyme, defined by an activity < 10%, through two main mechanisms: functional inhibition and accelerated clearance of the enzyme [31,32,33]. According to the most recent data in the literature, this severe deficiency, which is central to the pathophysiology of iTTP, results predominantly from functional inhibition, although there is considerable interindividual variability in the relative contribution of each mechanism [32]. Regardless of the predominant pathogenic pathway, the final consequence is the loss of enzymatic cleavage capacity.

In the absence of ADAMTS13 activity, ULvWF multimers persist uncleaved in the circulation. In the microcirculation (arterioles and capillaries), under conditions of extremely high shear stress, they undergo elongation, which triggers spontaneous platelet aggregation after a very short initial phase, on the order of milliseconds (approximately 10 ms) [34]. The massive consumption of platelets within these microthrombi generates severe thrombocytopenia, often with a platelet count < 20,000/µL. Simultaneously, red blood cells traversing the microcirculation, which is partially obstructed by these thrombi, are mechanically fragmented into schistocytes by forces that exceed the resistance of their membranes [35,36]. This results in MAHA, a setting in which elevated lactate dehydrogenase (LDH) levels are considered to primarily reflect tissue ischemia secondary to microvascular occlusions and, to a lesser extent, intravascular red blood cell lysis itself [37].

### 5.2. Relationship Between Microvascular Thrombosis and Organ Involvement

In iTTP, organ involvement does not result from the complete occlusion of a large-caliber vessel, as occurs in classical macrovascular thrombosis. Instead, the formation of thrombi within arterioles and capillaries produces a critical cumulative reduction in tissue perfusion. Through this multifocal and dynamic form of microvascular ischemia, several vascular territories are affected simultaneously, generating a heterogeneous clinical picture [2,38]. A fundamental characteristic of these microthrombi is their distinct composition, in experimental models of TTP, they have been described predominantly as platelet aggregates associated with ULvWF multimers, without a significant fibrin component or evident vascular injury. This characteristic suggests a mechanism of microvascular thrombosis that differs from the classical one and is dependent on the vWF-platelet interaction; it may occur spontaneously under conditions of blood flow and in the absence of traditional thrombotic risk factors [39].

Despite the systemic nature of the disease, certain organs, particularly the brain, heart, and gastrointestinal tract, appear to be preferentially and disproportionately affected, possibly due to the hemodynamic principles. These principles explain that the cleavage and elongation of ULvWF multimers are directly dependent on shear forces, with aggregation being preferentially triggered in vessels where shear rates exceed 2000 s^−1^ [28,40]. The cerebral, coronary, and splanchnic microcirculations are significant examples of vascular territories that meet these extreme biomechanical stress conditions [41]. Thus, the preferential formation of microthrombi at these sites explains why the initial clinical picture is dominated by acute neurological deficits, myocardial ischemia, and/or gastrointestinal symptoms in patients presenting to emergency departments [1].

The particular nature of these thrombi can confer an essential clinical property on the initial ischemic lesions: reversibility, which represents one of the main arguments in favor of the rapid initiation of treatment. Unlike the thrombi involved in the pathogenesis of classical thrombosis, in which fibrin networks and vascular injury predominate, microthrombi in TTP are predominantly platelet-rich and vWF-dependent, in experimental models of TTP, they have been described as having a limited fibrin component and no evident vascular injury, a feature that may favor the transient nature of microcirculatory dysfunction [39]. Thus, this particular architecture helps explain why microvascular occlusion may initially cause only hypoperfusion, resulting secondarily in tissue dysfunction, before irreversible structural infarction lesions develop [41]. A retrospective study conducted at a tertiary center showed that, among patients with thrombotic thrombocytopenic purpura and ADAMTS13 deficiency evaluated by magnetic resonance imaging (MRI), approximately 82% had acute cerebral abnormalities, while in nearly half of the cases (48%), the lesions actually represented reversible vasogenic edema—posterior reversible encephalopathy syndrome (PRES) [42]. However, the window of reversibility is temporally limited, and patients may deteriorate rapidly, with prolonged ischemia transforming reversible dysfunction into necrosis (irreversible organ damage) [43]. This rapid progression, with a fulminant course, justifies the need for recognition of the disease in the emergency setting and the initiation of specialized treatment to facilitate the resolution of these atypical microthrombi before irreversible organ damage develops [38].

## 6. Clinical Presenting Phenotypes in iTTP

### 6.1. Non-Specific and Prodromal Presentation

Despite the classical perception of iTTP as a hyperacute disease, the literature indicates that up to one-quarter of patients may experience vague, nonspecific, general prodromal symptoms even several weeks before diagnosis [18]. According to data from international registries, patients frequently report marked fatigue, fever (10–35% of cases), nausea and abdominal pain (35–39% of cases), and vomiting [1,18]. The Milan Registry, which monitored clinical presentations over a 14-year period, highlights the polymorphism of the clinical picture and provides a hierarchy of clinical manifestations at diagnosis. Thus, the most frequent manifestations are systemic symptoms (fever, fatigue, abdominal pain, vomiting, jaundice), occurring in approximately 72% of cases, closely followed by hemorrhagic manifestations (purpura, petechiae, ecchymoses, hematomas, gingival bleeding, gastrointestinal or respiratory tract bleeding, gross hematuria, and menometrorrhagia), occurring in 68% of cases, while renal (18%) and cardiovascular (10%) manifestations have a lower prevalence [6].

These initial nonspecific symptoms often contribute to diagnostic delays, with the literature emphasizing the frequent risk of attributing them to much more common conditions, such as transient ischemic attack, gastroenteritis, or sepsis [6,18]. In a retrospective study including 38 patients with thrombotic thrombocytopenic purpura, almost half of the cases (47%) had a delayed diagnosis, with a median delay of approximately 5 days [16]. Moreover, the main diagnostic confusion appears to occur with immune thrombocytopenia or another form of autoimmune cytopenia [18,26]. This confusion is related to a laboratory profile that may initially be misleading, as, at the first evaluation, patients with a delayed diagnosis had normal or only mildly decreased hemoglobin levels compared with those diagnosed early, while the schistocyte count could be below 1% or undetectable [16,26]. In this context, repeated examination of the peripheral blood smear over time becomes essential in patients with a high clinical suspicion, because schistocytes may be absent in the early stages of the disease [1].

Overall, these data highlight that diagnostic delay in iTTP is not due exclusively to the diagnostic difficulties of the disease, but rather to a much more complex process, dominated, on the one hand, by the nonspecific nature of disease onset and, on the other hand, by the variability of its clinical and laboratory expression. A study conducted in a cohort including 225 episodes in 143 patients reported that the median interval from symptom onset to the initiation of plasma exchange was 4.28 days, whereas the time from hospital presentation to treatment initiation was approximately 8.4 h [44], suggesting that most of the delay occurs before hospital presentation or during the initial stage of clinical recognition of the disease.

### 6.2. Core Hematologic Syndrome

Although the clinical presentation of thrombotic thrombocytopenic purpura was historically defined by the “classic pentad,” the contemporary definition of the disease now focuses exclusively on the recognition of the basic diagnostic dyad: MAHA and severe thrombocytopenia [4,45]. This constitutes the central hematological syndrome that dictates emergency therapeutic management.

From a laboratory perspective, at the time of initial presentation to medical care, the laboratory findings reveal the direct consequences of systemic microthrombosis: platelet consumption, mechanical intravascular hemolysis, and, variably, ischemic organ involvement [46]. Modern criteria define this hematological syndrome by the following characteristic abnormalities:

• Consumptive thrombocytopenia: It is usually profound, with the literature considering a platelet count < 30 × 10^9^/L to be typical [46]; in severe cases, values may be even lower, with a platelet count of 9 × 10^9^/L reported at presentation in a recent case of iTTP [3].
• Intravascular hemolysis: Decreased hemoglobin levels, with frequent moderate-to severe anemia, increased LDH and indirect bilirubin levels, reticulocytosis, decreased haptoglobin, and a negative Coombs test [1,45]
• Red blood cell morphology: The presence of schistocytes on the peripheral blood smear represents the defining morphological abnormality, with a threshold of ≥1% being considered suggestive of thrombotic microangiopathy in the absence of an alternative explanation [46].

Overall, this hematological dyad should be regarded as a laboratory warning sign in the emergency setting, rather than merely as a descriptive criterion of the disease. The association of profound thrombocytopenia with MAHA requires immediate suspicion of TTP, even in the absence of the classic pentad or evident organ involvement at presentation. Moreover, when the schistocyte count is below 1%, peripheral blood smear findings should be assessed over time and integrated into the overall clinical and laboratory context, without delaying treatment initiation whenever clinical suspicion is high [45,46].

### 6.3. Organ-Dominant Clinical Phenotypes

#### 6.3.1. Neurologic-Dominant Phenotype

Neurological involvement occupies a central place among the predominant presenting phenotypes of iTTP, both because of its frequency and its prognostic impact. Severe ADAMTS13 deficiency ultimately leads to microvascular platelet aggregation, causing organ ischemia, including cerebral ischemia [1,41]. Neurological symptoms should therefore not be interpreted as a secondary or nonspecific manifestation of the disease, but rather as an expression of cerebral microthrombosis.

The frequency of neurological manifestations at onset is high, and the neurological phenotype is polymorphic, ranging from apparently benign symptoms to severe encephalopathy, seizures, stupor, and coma [1,47,48]. Recent data from the literature report neurological manifestations in approximately 39–80% of patients with iTTP, including headache, confusion, seizures, or focal neurological deficits. In addition, in a French prospective cohort that included 130 patients, 108 (83%) presented with neurological signs at onset, the most frequent being headache (51%); limb weakness, paresthesia, and/or aphasia (49%); cognitive impairment or delirium (34%); pyramidal syndrome (30%); altered level of consciousness (20%); and visual symptoms (visual field deficits, reduced visual acuity, and floaters—20%) [47]. This clinical variability explains the difficulty of early disease recognition, particularly when the patient initially presents to an emergency department with suspected stroke or seizure and the hematological picture is incomplete.

A particular aspect is the “stroke-like” phenotype, in which iTTP mimics a primary neurovascular emergency. Cases have been described in which patients initially presented with dysarthria, hemiparesis, facial paralysis, aphasia, or altered level of consciousness, sometimes with normal brain imaging, but with hematological clinical and laboratory abnormalities, such as thrombocytopenia, MAHA, or severe ADAMTS13 deficiency, identified subsequently [25,49].

Neuroimaging has a fundamental role in characterizing cerebral involvement; however, it should not delay recognition of the underlying hematological syndrome. The lesions described may include acute cerebral ischemia, multifocal lesions, cerebral hemorrhage, and posterior reversible encephalopathy syndrome (PRES). In some situations, imaging performed in patients with neurological manifestations has identified acute central nervous system lesions in only a proportion of them, once again emphasizing that neurological assessment must be integrated with clinical and laboratory data. Likewise, normal imaging findings should not reduce the suspicion of iTTP when the clinical and laboratory context is suggestive.

Another important dimension of neurological involvement is its prognostic value. The presence of neurological manifestations from disease onset not only provides diagnostic guidance but may also indicate a more severe form of the disease and a higher risk of an unfavorable outcome. In the UK TTP Registry, a low Glasgow Coma Scale score at presentation was associated with a significant increase in mortality, whereas in the Spanish Registry, stupor or coma was independently associated with an increased risk of death [50,51]. Therefore, the severe neurological phenotype may justifiably be classified as a high-risk presentation that requires rapid assessment and prompt initiation of specific treatment.

In older patients, recognition of the neurological phenotype may be even more difficult. Data from the French literature indicate that patients aged at least 60 years may have an atypical neurological presentation, dominated by behavioral disturbances or delirium and accompanied by less severe cytopenias, resulting in a longer time to diagnosis [52].

Overall, the neurological phenotype of iTTP may represent one of the most important forms of presentation in the emergency setting. Any patient with unexplained neurological symptoms, particularly in the presence of thrombocytopenia, MAHA, or elevated LDH levels, should be rapidly evaluated for TTP.

#### 6.3.2. Cardiac Involvement and Biomarker Relevance

In the context of the systemic manifestations of iTTP/TTP, cardiac involvement may represent an organ-specific phenotype with an important impact on prognosis, but one that is often overlooked because myocardial injury may remain clinically silent. Compared with the neurological phenotype, in which the presentation appears to be dominated by clinical manifestations, cardiac involvement may initially be suggested only by ancillary investigation findings, particularly changes in troponin levels or nonspecific electrocardiographic abnormalities. This discrepancy between the severity of microvascular injury and its subtle clinical expression explains the need for cardiac assessment from the time of presentation [53,54].

Clinically, cardiac involvement may range from mildly symptomatic forms to severe or life-threatening manifestations, such as acute coronary syndrome, arrhythmias, heart failure, cardiogenic shock, or cardiac arrest. A retrospective study from the United States that included 15,054 hospitalizations for TTP reported cardiovascular complications in 25.3% of cases, including heart failure (8.3%), acute coronary syndrome (6.4%), atrial tachyarrhythmias (5.9%), ventricular tachyarrhythmias (2%), cardiac arrest (1.9%), and cardiogenic shock (0.5%). In addition, the same analysis found that the presence of a major cardiovascular complication was associated with significantly higher in-hospital mortality, approximately 19.7% compared with 4.1% in its absence [55]. This disease may also represent a rare cause of myocardial infarction in the absence of obstructive coronary artery disease; therefore, the diagnosis should be considered when signs of myocardial ischemia are associated with thrombocytopenia, MAHA, and schistocytes on the peripheral blood smear [56].

From a laboratory perspective, troponin, which is widely used as a biomarker, plays an essential role both as a marker of myocardial injury and as a prognostic indicator. In a study published in 2015 by Benhamou and colleagues, which included 133 patients with iTTP and severe ADAMTS13 deficiency, elevated troponin levels were observed in 59% of patients, more than half of whom had no evident clinical cardiac manifestations. In addition, in the same study, a cTnI level > 0.25 µg/L was identified as the only independent factor associated with death and/or treatment refractoriness and was associated with an approximately threefold increase in risk [53]. Similarly, in the UK TTP Registry, 68% of patients had elevated troponin levels at presentation, with an approximately sixfold higher mortality than patients with normal troponin levels [51]. In a more recent approach, Xiang and Dai proposed a prognostic stratification model based on the integration of information regarding myocardial injury, hemolysis, and renal dysfunction through the use of four biomarkers. In a retrospective cohort of 106 patients with TTP, elevated troponin (>353.1 pg/mL), LDH (>992 U/L), BUN (>10.9 mmol/L) and indirect bilirubin (>32.3 µmol/L) levels were associated with a progressive increase in the risk of 28-day mortality, with death rates ranging from 21.3% among patients with a score of 0–1 to 92% among those with a score of 4 (the score was calculated by the authors by assigning one point for each biomarker above the defined threshold) [57]. Accordingly, these data support the inclusion of troponin measurement in the initial evaluation of patients with iTTP, both to identify subclinical cardiac involvement and to estimate disease severity and early risk.

The cardiac phenotype should therefore be actively assessed in every patient with suspected TTP, even in the absence of a clinical presentation compatible with cardiac involvement. The initial evaluation should include a 12-lead electrocardiogram and troponin measurement, together with other investigations such as a complete blood count, peripheral blood smear, hemolysis markers, coagulation tests, and blood sampling for ADAMTS13 testing [19]. In practice, an elevated troponin level in a patient with severe thrombocytopenia and MAHA should not be interpreted exclusively as indicating a primary acute coronary syndrome, but rather as a possible expression of cardiac microthrombosis in TTP, with direct implications for patient triage, monitoring, and the initiation of specific emergency treatment.

#### 6.3.3. Abdominal and Gastrointestinal Phenotype

Abdominal and gastrointestinal manifestations represent a frequent but often underestimated component of the initial clinical presentation of iTTP. They are nonspecific, may precede recognition of the underlying hematological picture, and include symptoms such as abdominal pain, nausea, vomiting, and asthenia. Diarrhea may also occur as a clinical manifestation, but it is nonspecific and is not consistently reported as a predominant manifestation. Recent data summarized by Pishko and colleagues indicate that abdominal pain or nausea occurs in approximately 35–39% of patients with iTTP [1]. George describes gastrointestinal symptoms as common manifestations at onset, together with asthenia (weakness), purpura, and transient focal neurological deficits [58].

Clinically, the abdominal phenotype may range from nonspecific digestive symptoms to presentations that may mimic a primary abdominal emergency. Over the years, TTP has been described in the literature in association with several clinical entities, including severe abdominal pain, acute pancreatitis, acute acalculous cholecystitis, or postoperative settings, situations that may initially direct the evaluation and therapeutic management toward a digestive or surgical disease, while the decisive hematological findings may become evident later [59,60,61,62,63].

In everyday practice, the abdominal and gastrointestinal phenotype should be regarded as a form of iTTP presentation associated with a substantial risk of diagnostic confusion. In this context, the main diagnostic pitfall lies in attributing abdominal symptoms to conditions commonly encountered in ED, such as gastroenteritis, sepsis, or an acute abdomen, without considering the hematologic context of iTTP. Abdominal pain, nausea, vomiting, or diarrhea should not be interpreted in isolation, particularly in the presence of laboratory findings suggestive of TMA, such as thrombocytopenia, MAHA, elevated LDH, decreased haptoglobin, or schistocytes on the peripheral blood smear. Under these circumstances, investigations directed toward an abdominal cause should be accompanied by an active search for thrombotic microangiopathyto avoid delays in the diagnosis of iTTP.

#### 6.3.4. Renal Involvement in iTTP

Within the spectrum of thrombotic microangiopathies, renal involvement in iTTP occupies a particular position. Although it may be part of the clinical picture from disease onset, it is characteristically less severe and less frequently clinically dominant than in typical hemolytic uremic syndrome (HUS) and atypical/complement-mediated hemolytic uremic syndrome (aHUS), where renal involvement represents the predominant clinical feature [1,58,64]. Compared with HUS/aHUS, iTTP is much more frequently characterized by significant thrombocytopenia, MAHA, and neurological or cardiac involvement, while renal function is often normal or only mildly to moderately impaired. In everyday practice, this difference has significant practical relevance in the differential diagnosis between iTTP and HUS/aHUS, a distinction that ultimately may determine the initial therapeutic approach, including the indication for therapeutic plasma exchange versus complement-directed therapies [65,66,67].

However, renal involvement should not be considered absent in iTTP. In an analysis published by Page et al. based on Oklahoma data collected between 1995 and 2015, renal involvement was most often moderate: a creatinine level ≥ 1.5 mg/dL was documented in approximately 48% of patients, but only 14% had a creatinine level ≥ 2.5 mg/dL, while severe renal failure was reported in 5% of cases [23]. These data highlight that mild-tomoderate renal dysfunction may be observed in a substantial proportion of patients with iTTP, whereas severe acute kidney injury or dialysis-requiring renal failure is reported much less frequently. The same concept is supported by George and Nester, who emphasize that, although the disease may produce thrombi within the renal microvasculature, acute kidney injury and chronic kidney disease are uncommon. They explain this finding as probably being related to the predominant thrombotic mechanism, in contrast to other thrombotic microangiopathies in which parenchymal and endothelial renal injury appears to be more pronounced [58].

At the same time, data from studies available in the literature suggest that renal injury in iTTP may be underestimated. In a retrospective single-center cohort from France, which included 92 adult patients admitted to the intensive care unit with a confirmed diagnosis of TTP based on ADAMTS13 activity < 10%, Zafrani et al. reported acute kidney injury in 58.7% of patients, including severe forms, hematuria, hemoglobinuria, nephrotic-range proteinuria, and the need for renal replacement therapy [68]. In this context, although severe renal involvement is not a defining feature of iTTP, assessment of serum creatinine and urinalysis for the identification of proteinuria and hematuria remains important for assessing severity, monitoring the clinical course, and establishing the differential diagnosis. In addition, the development of severe renal failure or the need for dialysis should raise suspicion of another form of thrombotic microangiopathy and require the differential diagnosis to be revisited.

Another important aspect of renal function is its diagnostic value, as reflected by the inclusion of creatinine level in clinical scores used to estimate the likelihood of severe ADAMTS13 deficiency, such as the PLASMIC score and the French score [64,69]. Therefore, renal involvement should not be interpreted in isolation, but in the context of the entire clinical and laboratory picture, as the clinical distinction between TTP and HUS/aHUS may overlap [66]. Mild-to-moderate renal impairment is compatible with a diagnosis of iTTP, whereas severe renal failure, the predominance of renal manifestations, or the early need for dialysis should prompt the examining physician to reconsider the differential diagnosis toward HUS/aHUS or other forms of TMA [65,67,70].

### 6.4. Mixed and Atypical Presentation

Although the analysis of predominant organ phenotypes is useful for systematizing the clinical presentation, in everyday emergency department practice, iTTP frequently presents as a multisystem disease in which neurological, cardiac, renal, digestive, and hematological involvement may overlap to form the patient’s clinical picture. In an analysis conducted by Mariotte et al., which ultimately included 772 patients, iTTP was associated with various clinical contexts in approximately 51% of cases, including autoimmune diseases such as systemic lupus erythematosus or antiphospholipid syndrome, infections, pregnancy, malignancies, transplantation, or drug exposure. In this way, a mixed presentation is not an exception, but rather an expression of the systemic nature of the disease [71].

A particular group of atypical forms is represented by iTTP associated with autoimmune diseases, especially systemic lupus erythematosus (SLE). In these cases, the clinical picture may be difficult to distinguish from the activity of the underlying disease, other thrombotic microangiopathies, or antiphospholipid syndrome [72,73]. Clinical data available in the literature indicate that patients with SLE may present with less evident clinical signs when TTP is also present, requiring a lower threshold of clinical suspicion to avoid delaying treatment initiation [73]. In addition, some SLE-associated forms may be refractory to therapeutic plasma exchange and corticosteroid therapy, requiring second-line or biological therapies [72]. Diagnostic complexity is further increased by the fact that, in patients with SLE, the occurrence of TTP has been associated with increased disease activity, elevated total cholesterol levels, and renal involvement [74].

Another category at risk of atypical presentation is represented by older patients. In the French cohort reported by the Reference Center for Thrombotic Microangiopathies, patients older than 60 years had a longer interval from hospital admission to diagnosis, a clinical picture dominated by atypical neurological manifestations such as delirium or behavioral disturbances, renal involvement, higher troponin levels, but less profound cytopenias compared with younger patients. These characteristics are associated with an unfavorable prognosis, with age over 60 years, cardiac involvement, and elevated creatinine being associated with early mortality [52].

Overall, mixed and atypical forms highlight that recognition of iTTP in the emergency department may become a genuine challenge, because patients do not always present with the classic clinical picture. The disease may begin in a context that alters its clinical expression, as in older patients, those with associated autoimmune diseases, or those with comorbidities that provide alternative explanations for cytopenias and organ dysfunction. Therefore, in atypical forms, rapid collection of samples for ADAMTS13 testing and continued suspicion of thrombotic microangiopathy remain essential when the clinical and laboratory profile is suggestive.

The dominant presenting phenotypes, common diagnostic mimics, and key clinical clues discussed above are summarized in Table 1.

## 7. Diagnostic Approach in the Emergency Setting

### 7.1. Initial Clinical Suspicion: When to Consider iTTP

The central issue during the initial presentation to the emergency department is not the recognition of a “complete” form of the disease, but the identification of the point at which TTP/iTTP should be actively considered in the differential diagnosis. The threshold for initial clinical suspicion should remain low because the disease is rare but rapidly progressive and potentially fatal, while its presentation is often incomplete and nonspecific. The classic pentad is present at onset in only a small proportion of patients; therefore, suspicion should be raised promptly in the presence of severe thrombocytopenia and MAHA, even in the absence of fever, neurological involvement, or renal dysfunction [11,19]. Rapid recognition remains essential because diagnostic delay leads to delayed initiation of specific treatment in a disease whose prognosis depends significantly on early intervention [4,75].

In current practice, clinical suspicion should be raised by the association of severe, unexplained thrombocytopenia with other findings suggestive of MAHA and/or ischemic organ involvement. Moreover, it should be emphasized that this suspicion should not depend on the immediate availability of the ADAMTS13 result or on the identification of a high schistocyte count on the initial peripheral blood smear, because therapeutic decisions often need to be made before these results become available, and schistocytes may be absent or present in low numbers at the first evaluation [11,16]. In cohorts of patients admitted to intensive care units, severe thrombocytopenia may have important discriminatory value in directing the diagnosis toward TTP among thrombotic microangiopathies, supporting its important role as a warning sign [76].

An important obstacle to the early diagnosis of this disease remains diagnostic anchoring on a more common or apparently more plausible explanation, such as immune thrombocytopenia or other autoimmune cytopenias, sepsis, gastroenteritis, or an acute neurological event [18,26]. Therefore, clinical suspicion should prevail over the isolated interpretation of a single laboratory finding, and evaluation for iTTP should be initiated promptly when the clinical and laboratory profile suggests thrombotic microangiopathy.

### 7.2. Initial Laboratory Evaluation and Guidance of the Differential Diagnosis

The initial laboratory evaluation of a patient with suspected TTP/iTTP must be rapid, targeted, and interpreted in an integrated manner. The main objective is not the early confirmation of the diagnosis before treatment initiation, but the identification of a thrombotic microangiopathy profile that justifies blood sampling for ADAMTS13 testing and the initiation of the appropriate therapeutic management. During the initial evaluation, severe thrombocytopenia represents a significant laboratory finding, and the threshold of <30 × 10^9^/L is included even in the clinical scores used to estimate the probability of severe ADAMTS13 deficiency, such as the PLASMIC and French scores [69,77]. Platelet counts < 20 × 10^9^/L may also represent a useful laboratory clue in directing the clinician toward TTP, although this finding is not specific in the absence of the other features of thrombotic microangiopathy [78]. The complete blood count must be interpreted as a whole, through a careful analysis that also includes red blood cell indices and the reticulocyte count, because a discordant profile may suggest clinical entities that mimic iTTP. In iTTP, anemia is usually normocytic, and reticulocytosis supports its hemolytic nature; conversely, marked macrocytosis or an inadequate reticulocyte response relative to the degree of anemia should prompt reconsideration of the differential diagnosis, including disorders of cobalamin metabolism [79,80,81,82].

Laboratory evidence of MAHA is based on the association of elevated LDH, elevated indirect bilirubin, decreased or undetectable haptoglobin, reticulocytosis, and a negative direct Coombs test [1]. Particular attention should be paid to the interpretation of LDH levels because, although LDH is a marker of hemolysis, in iTTP, elevated LDH levels andfailure to markedly reduce LDH by day 5 have been associated with mortality in iTTP disease severity, making correlation with signs of organ involvement necessary [83]. In parallel, coagulation tests are useful for the differential diagnosis. Unlike disseminated intravascular coagulation, in which more significant abnormalities in PT, INR, fibrinogen, and D-dimer levels are expected, in iTTP, PT and aPTT are usually normal or only mildly altered [78,79].

The peripheral blood smear is an essential investigation in the initial evaluation, as the presence of schistocytes supports the diagnosis of thrombotic microangiopathy. The ICSH recommendations consider a schistocyte percentage > 1% to support thrombotic microangiopathy in adults in the absence of alternative explanations. However, the peripheral blood smear should not represent an absolute exclusion criterion because schistocytes may be absent or insufficiently represented at the initial evaluation, requiring repeated assessment over time when clinical suspicion is high. In addition, schistocyte quantification is subject to interobserver and interinstitutional variability, making it necessary to correlate the result with the platelet count, hemolysis markers, coagulation tests, and signs of organ involvement [84,85].

In parallel with the initial laboratory evaluation, when clinical suspicion is high, blood samples should be collected for the measurement of ADAMTS13 activity and, whenever possible, for the detection of ADAMTS13 inhibitors or anti-ADAMTS13 antibodies. These samples must be collected before plasma administration or therapeutic plasma exchange because treatment may alter the interpretation of the results. ADAMTS13 activity < 10% supports the diagnosis of TTP, while the demonstration of an inhibitor or anti-ADAMTS13 antibodies supports the acquired immune form of the disease. It should be emphasized that ADAMTS13 testing has important practical limitations: it is not immediately available in all centers, laboratory methods differ, and the result must be interpreted in correlation with the patient’s clinical and laboratory findings. Therefore, the immediate unavailability of the ADAMTS13 result should not delay the initiation of appropriate therapeutic management when clinical suspicion of this disease is high [78,86].

### 7.3. Pretest Probability Scores

In patients with suspected iTTP, clinical probability scores estimate the pretest probability of severe ADAMTS13 deficiency before the test result becomes available. Their utility is therefore greatest in emergency departments and during the first hours after admission, as they contribute to the rapid stratification of clinical probability and guide the decision to initiate treatment based on the first available clinical and laboratory data. The ISTH guidelines recommend assessing pretest probability through clinical judgment and/or the use of risk models, such as the PLASMIC and French scores. It should be noted that these scores do not confirm the diagnosis and do not replace ADAMTS13 testing but, as decision-support tools, help guide initial management promptly in patients with MAHA and thrombocytopenia [12,69].

The PLASMIC score is a composite tool consisting of seven binary variables, with one point assigned to each: platelet count < 30 × 10^9^/L, evidence of hemolysis, absence of active cancer, absence of solid-organ or stem-cell transplantation, MCV < 90 fL, INR < 1.5, and serum creatinine < 2 mg/dL. It was developed by Bendapudi et al. based on a cohort of 214 adult patients with thrombotic microangiopathy and suspected TTP who were evaluated at three academic centers in Boston. The total score ranges from 0 to 7 and allows patients into three probability categories for severe ADAMTS13 deficiency: low risk for total scores between 0 and 4, intermediate risk for a score of 5, and high risk for scores of 6–7. This stratification correlated with the frequency of severe ADAMTS13 deficiency, which was absent in patients with scores between 0 and 4, present in 5% of those with a score of 5, and present in 81% of those with scores of 6–7 [69]. The practical value of the score therefore lies in the rapid assessment of diagnostic probability: low scores make TTP less likely, whereas scores of 6–7 support a high probability, without confirming the diagnosis in the absence of ADAMTS13 testing. This interpretation is also supported by the meta-analysis conducted by Paydary et al., which included 13 studies and 970 patients: a PLASMIC score ≥ 5 had a sensitivity of 99% but a moderate specificity of 57%, making it useful as a screening tool rather than a confirmatory test [87]. In the external validation conducted by Li et al., high-risk scores, defined as 6–7, predicted severe ADAMTS13 deficiency with a sensitivity of 90%, a specificity of 92%, and a positive predictive value of 72%, whereas classification in the 0–5 group had a negative predictive value of 98% [64].

A major limitation in the practical use of the PLASMIC score is that it must be applied under the conditions for which it was developed: adults with clinically suspected TTP, low platelet counts, and MAHA, generally with schistocytes present on the peripheral blood smear [69]. Its application in the absence of these clinical and laboratory prerequisites may be misleading and may compromise its clinical usefulness for risk assessment. Furthermore, the score should be used with considerable caution when likely or evident alternative causes of TMA are present, as its performance may differ in populations other than the one in which it was developed. The result should be interpreted alongside clinical and laboratory data and, whenever possible, ADAMTS13 activity. Another limitation is that, in older patients, the sensitivity of the score decreases, partly because of less profound thrombocytopenia and higher serum creatinine levels, which reduce the probability of classification in the high-risk category. Liu et al. showed that the sensitivity of a PLASMIC score ≥ 5 decreased from 91.4% (95% CI, 76.9–98.2%) in the 18–39 year age group to 76.9% (95% CI, 42.6–95.0%) in patients aged ≥ 60 years. Furthermore, in cohorts of patients admitted to intensive care units, the performance of the score was lower than that of the French score and even that of severe thrombocytopenia used alone, with an AUC of 0.67 (95% CI, 0.58–0.76), compared with 0.86 (95% CI, 0.81–0.92) [76]. Therefore, a low or intermediate PLASMIC score should not exclude iTTP when clinical suspicion is high, particularly in atypical forms, older patients, intensive care units, the presence of possible alternative causes of TMA, or in clinical contexts in which the performance of the score has not been sufficiently validated [76,88].

The French score, also known as the Coppo score, is a simpler tool than the PLASMIC score and was developed using data from the French Reference Center for Thrombotic Microangiopathies. In the original study, Coppo et al. identified three variables associated with severe acquired ADAMTS13 deficiency: platelet count < 30 × 10^9^/L, serum creatinine ≤ 200 μmol/L, and the presence of antinuclear antibodies (ANA). The presence of at least one of these criteria had a sensitivity of 98.8% (95% CI, 96.9–100%) and a specificity of 48.1% (95% CI, 38.9–59.3%) for severe acquired deficiency of the enzyme [77]. Although ANA was part of the initial model, severe thrombocytopenia and creatinine ≤ 200 μmol/L had the strongest association with ADAMTS13 deficiency, and the use of these two criteria resulted in a low number of misclassifications [77]. The French score was subsequently also applied in a simplified form without ANA, based on two criteria available at presentation: platelet count < 30 × 10^9^/L and creatinine ≤ 2.26 mg/dL [88,89].

Its main limitations include the fact that it is not a standalone diagnostic test, its limited specificity, and the need to exclude evident alternative or secondary causes of TMA, such as active cancer, transplantation, and severe sepsis. Regarding its use in intensive care units, it showed good performance in differentiating TTP from other thrombotic microangiopathies, with an AUC of 0.86, superior to the PLASMIC score but similar to the isolated use of severe thrombocytopenia [76]. However, as with the PLASMIC score, its performance decreases in older patients. The sensitivity of a French score ≥ 1 was 100% in the 18–39-year age group, 96.2% in those aged 40–59 years, and 76.9% in patients aged ≥ 60 years, whereas for a French score ≥ 2, sensitivity was 72.2% in the 18–39 year age group, 61.5% in those aged 40–59 years, and 46.2% in patients aged ≥ 60 years [88].

Figure 1 presents a side-by-side comparison of the PLASMIC and French scores, highlighting their score-estimated probability categories for rapid bedside interpretation.

### 7.4. Integration of Clinical and Laboratory Data in Emergency Decision Making

In the emergency setting, diagnostic suspicion of iTTP should not be approached as a linear sequence of confirmations, but as a complex process that integrates, in parallel, the clinical picture, complete blood count, hemolysis markers, peripheral blood smear, exclusion of alternative causes, and clinical probability scores. The ISTH guidelines recommend estimating the pretest probability through clinical assessment and/or models such as the PLASMIC or French scores, together with blood sampling for ADAMTS13 activity measurement and testing for an inhibitor or anti-ADAMTS13 antibodies [12]. In the same direction, the algorithm proposed by Pishko et al. in 2025 begins with the identification of patients with newly developed thrombocytopenia and anemia, confirmation of the laboratory profile through specific laboratory tests, and exclusion of alternative etiologies before calculating the clinical score for risk stratification and guidance toward the appropriate therapeutic management. In the emergency department, this entire process must be supported by a set of investigations that outline the patient’s initial profile, confirm the presence of thrombotic microangiopathy, and exclude alternative causes: complete blood count, peripheral blood smear, hemolysis markers, coagulation tests, renal and liver function tests, blood sampling for ADAMTS13 testing, and assessment of organ involvement according to the clinical context [1,16].

The practical threshold for therapeutic decision making may be reached when the patient has a profile of probable thrombotic microangiopathy, in the absence of an evident secondary cause and with at least an intermediate clinical risk of TTP. In this situation, hematology consultation should be requested and treatment initiated promptly on the basis of the available clinical and laboratory data, without being delayed until the ADAMTS13 results become available [90]. In modern practice, treatment with the monoclonal antibody caplacizumab may be initiated either on the basis of clinical suspicion supported by probability scores or after rapid confirmation of severe ADAMTS13 deficiency, depending on the availability of testing and the center’s protocol [89]. If the initial peripheral blood smear is inconclusive, the examination should be repeated over time; however, the absence of complete morphological confirmation at the first evaluation should not invalidate the clinical suspicion of iTTP when the clinical and laboratory profile is suggestive [91].

As a life-threatening disease, the time dimension is essential: the integration of clinical and laboratory data has practical value only if it rapidly leads to the initiation of therapeutic management. Sawler et al. emphasize that guidelines recommend initiating therapeutic plasma exchange within the first 4–8 h after diagnostic suspicion is raised; however, in real-world practice, this objective is difficult to achieve. In their cohort, the median time from suspicion to the first plasma exchange was 10.7 h, with 59% of episodes exceeding the 8 h threshold [92]. Other studies have shown that treatment initiation may also be delayed by other causes, including diagnostic uncertainty, interhospital transfer, and the availability of apheresis services [27,93]. At the same time, later initiation of plasma exchange has been associated with less favorable clinical outcomes in some cohorts [15,93]. Therefore, the emergency decision should be directed toward early activation of the therapeutic pathway, without waiting for all diagnostic elements to be complete.

Figure 2 translates this approach into a practical algorithm intended to support the emergency clinician. It integrates recognition of the TMA profile, the need to exclude obvious secondary causes, early blood sampling for ADAMTS13 testing concomitantly with the request for hematology consultation, and, finally, risk stratification to guide appropriate management using the PLASMIC or French scores to estimate pretest probability. The differential diagnosis should include the major cause of MAHA or secondary TMA: severe sepsis, disseminated intravascular coagulation (CID), HUS/STEC-HUS (Shiga toxin-producing Escherichia coli hemolytic-uremic syndrome)/aHUS, malignant hypertension, pregnancy-related microangiopathies, and TMA associated with transplantation, drugs, or malignancy [67,68,70]. However, the evaluation and exclusion of these causes should not delay the initiation of treatment when the probability of iTTP is high.

In addition to the algorithm, Figure 3 summarizes the main practical messages adapted for the emergency physician. It brings together information on the laboratory red flags suggestive of iTTP, priority actions, the critical time threshold, and a checklist of alternative diagnoses that should be rapidly ruled out.

## 8. Critical Appraisal of Current Clinical Guidelines

### 8.1. Guideline Convergence and Implementation Barriers in Emergency Care

Current guidelines agree that iTTP is a hematologic emergency in which treatment should not be delayed when clinical suspicion is high. In this regard, the ISTH recommendations for the treatment of TTP place therapeutic plasma exchange at the center of acute management and support the early addition of corticosteroid therapy, starting from presentation. Recent British studies also describe a similar approach, in which therapeutic plasma exchange, corticosteroid therapy, and specific adjunctive therapies are integrated early into therapeutic management [12,45]. Although ADAMTS13 activity < 10% remains the defining element for confirming the diagnosis in an appropriate clinical context, from an emergency medicine perspective, the practical relevance lies in supporting rapid intervention when confirmation is not yet available during the critical period for initial decision making. Although certain nuances between guidelines may be identified regarding the exact timing of specific immunomodulatory therapies, the main message for the emergency physician remains the need to initiate plasma exchange immediately [90,94,95,96].

From the perspective of emergency medicine, the main limitation of the guidelines is their dependence on resources that are not widely available in every emergency department: rapid ADAMTS13 testing, continuous availability of therapeutic plasma exchange, immediate hematological expertise, or access to immunomodulatory therapies. In real-world practice, the sample for ADAMTS13 testing is frequently sent to specialized laboratories, and the turnaround time may exceed the window of opportunity within which the initial therapeutic decision must be made. Rapid ADAMTS13 assays represent a promising direction, with some results being available in less than one hour and showing very good performance compared with standard tests; however, their widespread implementation remains dependent on local infrastructure and does not completely eliminate the need for empirical decision making in cases with high clinical suspicion [19,97,98].

In everyday practice, the application of these clinical protocols is frequently limited by logistical obstacles and the organization of the healthcare system. In this regard, the national HaemSTAR audit conducted in the United Kingdom showed that increasing the local availability of therapeutic plasma exchange increased the proportion of patients treated within the first 8 h, emphasizing the essential role of infrastructure in appropriate case management [27]. Furthermore, in a cohort including 1096 patients with severe forms, Van de Louw et al. demonstrated that the initiation of therapeutic plasma exchange upon admission to the intensive care unit or within the first day after admission was independently associated with reduced mortality [15]. In this way, the main problem is not exclusively the existence of guidelines, but how they can be translated into current practice through the organization of functional emergency pathways to ensure appropriate management: local triage protocols, early recognition of the disease, immediate availability of hematology consultation, blood sampling for ADAMTS13 testing, availability of therapeutic plasma exchange, and clear transfer protocols to experienced centers.

### 8.2. Knowledge Gaps and Future Directions

Despite recent scientific advances regarding this absolute hematological emergency, one of the main gaps remains the absence of diagnostic pathways specifically validated for use in emergency departments. The clinical scores currently available are of real value; however, they were developed or validated predominantly in cohorts of patients with suspected thrombotic microangiopathy or TTP, rather than in the general population presenting to the emergency department with anemia, thrombocytopenia, and/or nonspecific symptoms [19,87]. This limitation is particularly relevant because iTTP is a rare disease with a polymorphic clinical picture that may easily mimic other common emergencies, in which recognition depends on the rapid definition and integration of clinical and laboratory data rather than on the presence of a single defining sign. For this reason, future research directions should include the prospective validation of triage algorithms adapted to emergency medicine, capable of identifying high-risk patients early without leading to the unjustified overuse of empirical therapy.

A second important gap concerns the performance of current clinical scores in special populations and particular or complex clinical contexts. The PLASMIC score represents a valuable screening tool; however, the existing data are derived from retrospective studies or populations selected on the basis of suspected TTP, and prospective evaluation remains necessary [87]. In addition, in older patients, both the PLASMIC and French scores have lower sensitivity and specificity, probably because of less severe thrombocytopenia and the higher frequency of renal involvement in this population [88]. Therefore, these scores should be understood and used as decision-support tools, but they cannot be used to exclude the diagnosis. Studies are needed to evaluate their performance both in emergency departments and in specific patient populations, including older patients and those with sepsis, malignancies, autoimmune diseases, secondary forms of thrombotic microangiopathy, or post-transplant conditions.

A major direction for development is the widespread availability of rapid ADAMTS13 testing. At present, standard assays for ADAMTS13 activity are frequently available only in specialized laboratories, and the result is often available with a delay of at least one day, which maintains reliance on empirical clinical decision making during the initial phase [97]. Rapid assays have demonstrated promising analytical performance and turnaround times of less than one hour; however, they must be integrated into validated clinical algorithms because ADAMTS13 results cannot be interpreted in isolation, particularly in cases with a low clinical probability or in special situations [97,98,99]. Their implementation could certainly facilitate diagnosis in the emergency setting, but it remains dependent on several factors, including availability, cost, standardization, and, not least, interpretation within the clinical context.

Last but not least, a key remaining gap is organizational, concerning how clinical suspicion is rapidly translated into a functional diagnostic and therapeutic pathway. Diagnostic uncertainty, interhospital transfer, or the absence of a clear local pathway. Studies assessing the time to therapeutic plasma exchange have shown that the recommended intervals are difficult to achieve in current practice, although they remain an essential objective. It should nevertheless be noted that the implementation of standardized institutional pathways for TTP, coordinated by dedicated teams, has been associated with improved coordination of care, increased use of modern therapies, and reductions in relapses or deaths; however, the available data currently come from a single-center project involving a small number of episodes and several interventions applied simultaneously [100].

## 9. Conclusions

In essence, iTTP should be regarded as a complex hematological emergency with an extremely narrow therapeutic window, in which the diagnosis must be suspected before it is confirmed. For the emergency physician, the association between thrombocytopenia and MAHA should represent a warning sign, particularly when neurological, cardiac, gastrointestinal, or systemic manifestations may mimic a much more common emergency. The emergency physician should contact the hematologist and activate the therapeutic pathway whenever clinical suspicion is high.

Optimal management of iTTP extends beyond the expertise of a single specialty and depends on the rapid functioning of a network involving the emergency department, hematology, the laboratory, the intensive care unit, and specialized centers. Future directions, such as rapid ADAMTS13 testing, emergency-adapted triage algorithms, and personalized therapies, may reduce uncertainty, but they do not replace the essential principle of current practice: in a patient with thrombocytopenia, evidence of MAHA, and organ involvement, iTTP should be treated as a probable diagnosis and a therapeutic emergency, rather than as a rare hypothesis to be confirmed at a later stage.

## Figures and Tables

**Figure 1:**
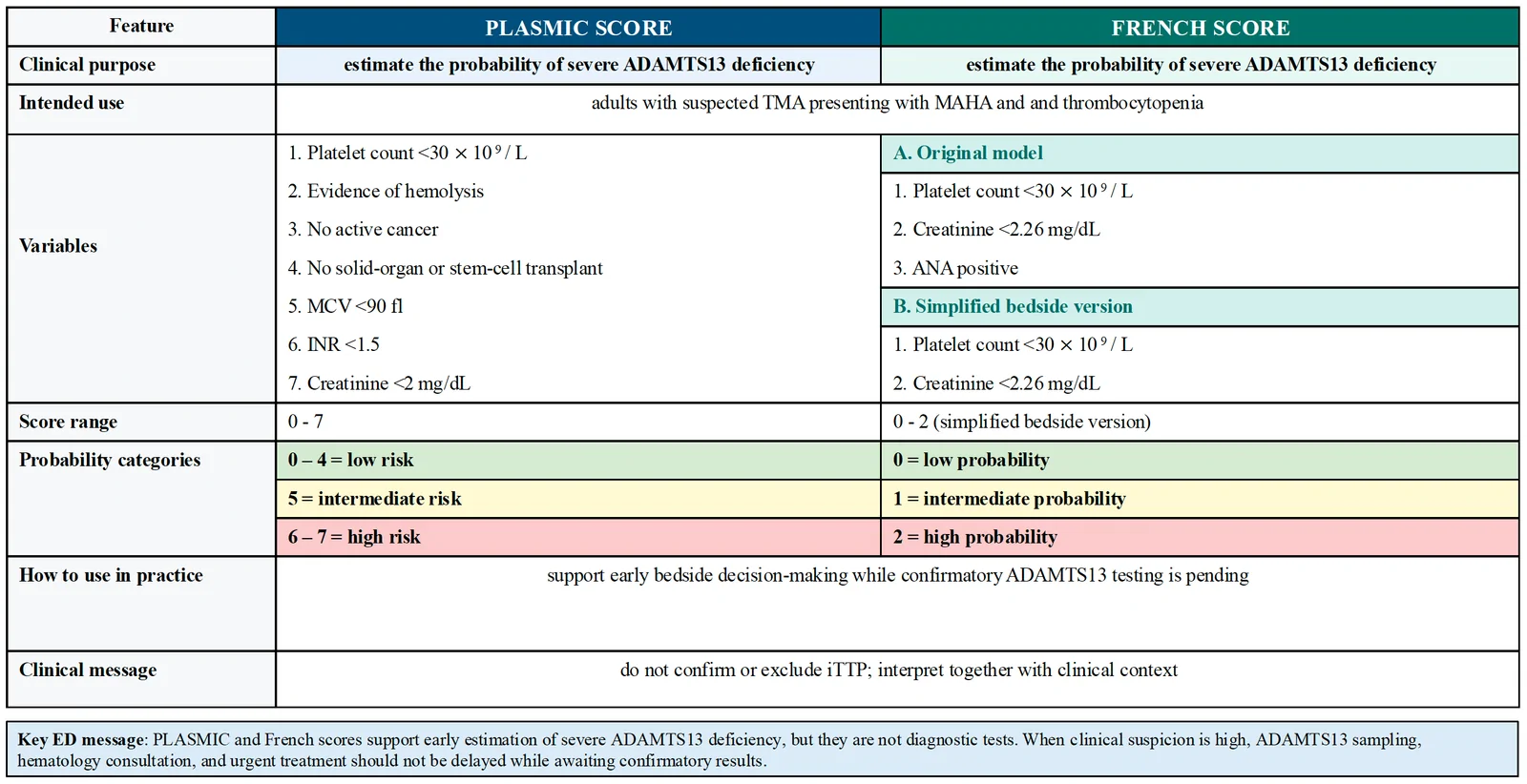
Side-by-side comparison of the PLASMIC and scores for estimating the probability of severe ADAMTS13 deficiency in suspected iTTP (Abbreviations: TMA, thrombotic microangiopathy; iTTP, immune thrombotic thrombocytopenic purpura; MCV, mean corpuscular volume; INR, International Normalized Ratio; ANA, antinuclear antibody; ED, Emergency Department).

**Figure 2:**
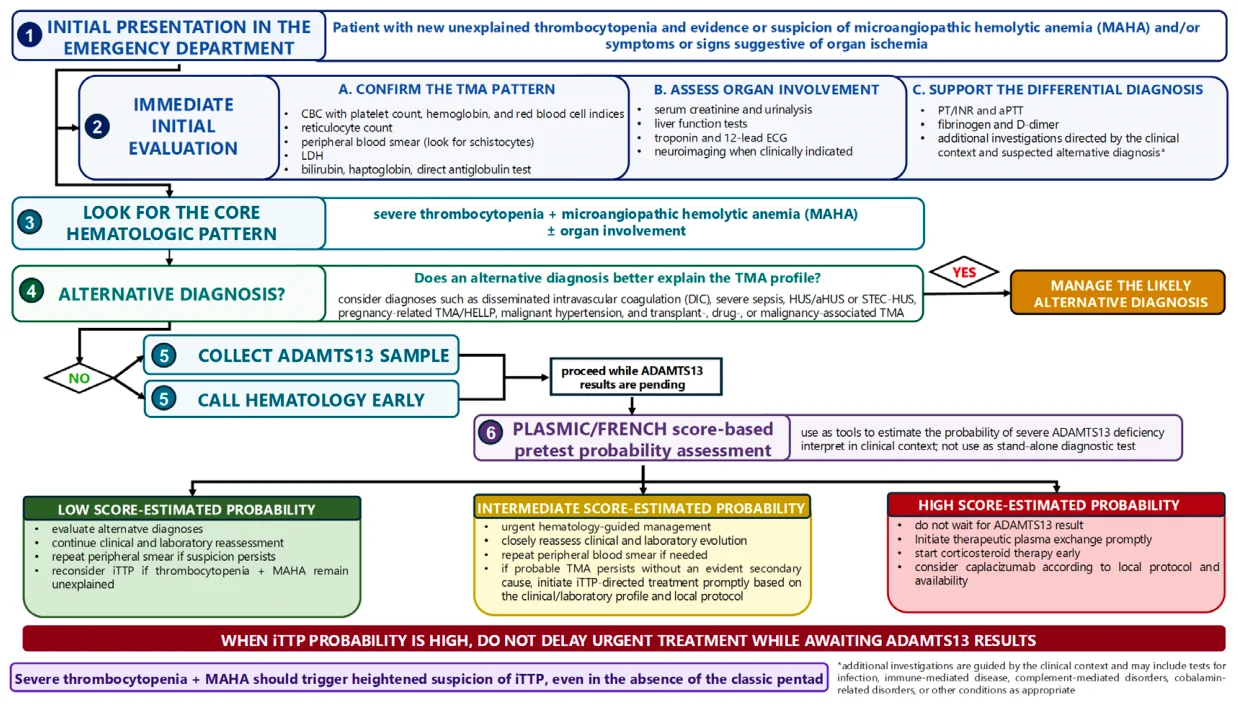
Stepwise emergency department algorithm for suspected TTP/iTTP—from early recognition to treatment activation. ADAMTS13 sampling, hematology consultation, probability assessment, and treatment activation should proceed according to clinical context and local protocols (Abbreviations: iTTP, immune thrombotic thrombocytopenic purpura; MAHA, microangiopathic hemolytic anemia; CBC, complete blood count; LDH, lactate dehydrogenase; PT, prothrombin time; INR, International Normalized Ratio; aPTT, activated partial thromboplastin time; HUS, hemolytic uremic syndrome; aHUS, atypical hemolytic uremic syndrome; STEC-HUS, Shigatoxin-producing Escherichia coli hemolytic uremic syndrome).

**Figure 3:**
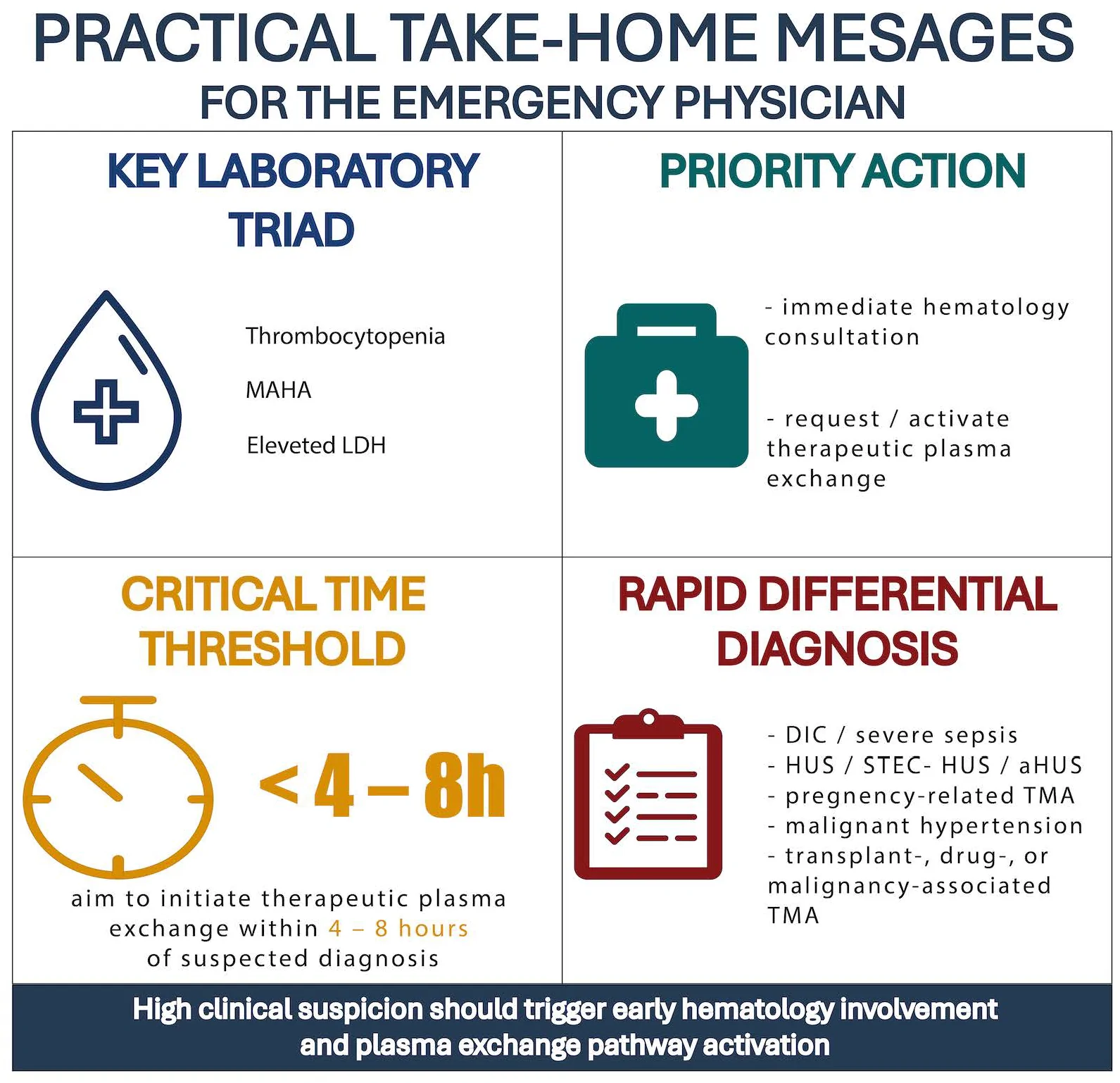
Practical take-home messages for the emergency physician in suspected iTTP (Abbreviations: iTTP, immune thrombotic thrombocytopenic purpura; MAHA, microangiopathic hemolytic anemia; LDH, lactate dehydrogenase; DIC, disseminated intravascular coagulation; HUS, hemolytic uremic syndrome; aHUS, atypical hemolytic uremic syndrome; STEC-HUS, Shigatoxin-producing Escherichia coli hemolytic uremic syndrome; TMA, thrombotic microangiopathy).

**Table 1:** Dominant presenting phenotypes, common diagnostic mimics, and key clinical clues suggesting iTTP.

Presenting Phenotype	Main Clinical Manifestation	Common Diagnostic Mimics	Key Clues Suggesting iTTP
Neurological	headache, confusion, seizures, focal neurological deficits, altered consciousness; stroke-like presentations	• ischemic stroke • transient ischemic attack • seizure disorder	• thrombocytopenia and MAHA • neurological abnormalities may fluctuate; • normal initial neuroimaging does not exclude iTTP
Cardiac	troponin elevation, ECG abnormalities, acute coronary syndrome, arrhythmias, heart failure	• acute coronary syndrome • other primary cardiac conditions	• myocardial injury associated with thrombocytopenia and MAHA; • cardiac involvement may be clinically silent
Abdominal/ Gastrointestinal	abdominal pain, nausea, vomiting, diarrhea; occasionally acute-abdomen presentations	• gastroenteritis • pancreatitis • acute abdomen • acute acalculous cholecystitis • other surgical conditions	• gastrointestinal symptoms may precede recognition of the hematological syndrome; • concomitant thrombocytopenia/MAHA should prompt consideration of iTTP
Renal	usually mild-to-moderate renal impairment; hematuria and proteinuria may occur	• HUS/aHUS • other thrombotic microangiopathies	• mild-to-moderate renal involvement is compatible with iTTP; • severe renal failure, predominant renal involvement, or early dialysis requirement should prompt reconsideration of the differential diagnosis
Mixed/ Atypical	overlapping neurological, cardiac, renal, gastrointestinal, and systemic manifestations; atypical presentations in older patients or those with autoimmune disease	• sepsis • autoimmune cytopenias • underlying autoimmune disease • other TMAs	• absence of the classic presentation should not lower suspicion when thrombocytopenia and MAHA coexist with otherwise unexplained organ dysfunction

Authors’ synthesis based on the clinical evidence discussed in the text. Abbreviations: iTTP, immune thrombotic thrombocytopenic purpura; MAHA, microangiopathic hemolytic anemia; ECG, electrocardiogram; HUS, hemolytic uremic syndrome; aHUS, atypical hemolytic uremic syndrome; TMAs, thrombotic microangiopathies.

## Data Availability

No new data were created or analyzed in this study. Data sharing is not applicable to this article.
